# Can Adsorption on Graphene be Used for Isotopic Enrichment? A DFT Perspective

**DOI:** 10.3390/molecules23112981

**Published:** 2018-11-15

**Authors:** Mateusz Pokora, Piotr Paneth

**Affiliations:** Institute of Applied Radiation Chemistry, Lodz University of Technology, Zeromskiego 116, 90-924 Lodz, Poland; 205841@edu.p.lodz.pl

**Keywords:** DFT, graphene, ONIOM, isotope effect

## Abstract

We have explored the theoretical applicability of adsorption on graphene for the isotopic enrichment of aromatic compounds. Our results indicate that for nonpolar molecules, like benzene, the model compound used in these studies shows a reasonable isotopic fractionation that is obtained only for the deuterated species. For heavier elements, isotopic enrichment might be possible with more polar compounds, e.g., nitro- or chloro-substituted aromatics. For benzene, it is also not possible to use isotopic fractionation to differentiate between different orientations of the adsorbed molecule over the graphene surface. Our results also allowed for the identification of theory levels and computational procedures that can be used for the reliable prediction of the isotope effects on adsorption on graphene. In particular, the use of partial Hessian is an attractive approach that yields acceptable values at an enormous increase of speed.

## 1. Introduction

Properties of newly developed carbon materials, like fullerenes, nanotubes, and graphene, are getting increasing attention due to scientific curiosity but also due to possible practical applications [1]. With its high conductivity both electrical [2] and thermal [3], graphene has been studied as a possible material for transistors and other electrical appliances [4]. The large surface area makes graphene a potentially perfect sorbent. Therefore its sorption properties are of great interest and it is thus not surprising that they were studied extensively both experimentally and theoretically. Graphene was examined as a potential sorbent of organic pollutants, such as dyes [5], aromatic compounds [6,7,8], and heavy metals [5,9] from water and aqueous solutions. Furthermore, it was considered for the capturing of biologically active substances [8,10] as well as a potential “scavenger” of greenhouse gases like CO_2_ [11,12,13,14], CH_4_, or N_2_ [12]. It has been reported that the adsorption of small molecules like H_2_O, H_2_, O_2_, CO, NO, and NO_2_ leads to the doping of graphene [15] which makes it a useful method for creating potential graphene-based electronic components.

Although the price of graphene and its synthesis continue to drop [16], studies on large surface areas of graphene are still expensive. This is where theoretical studies come in: molecular dynamics and Monte Carlo simulations were used to investigate the process of the adsorption of diatomic halogen molecules F_2_, Cl_2_, Br_2_, I_2_ [17], and neutral aromatic pollutants [18]. Density functional theory studies were used to examine the thermodynamics and kinetics of the absorption of CH_4_ [19], O_3_ [20], small molecules, both inorganic (N_2_, CO_2_, H_2_, and Ar) [21,22] and organic (acetone, acetonitrile, dichloromethane, ethanol) [23,24], as well as polycyclic aromatic hydrocarbons [25,26] and aromatic compounds [27,28,29,30,31,32].

Continuing our interest in the experimental and theoretical scrutiny of isotope effects we have turned our interest to the isotopic fractionation associated with adsorption of aromatic compounds on graphene, which might potentially find practical applications in obtaining isotopically enriched materials. There are two interesting problems associated with the theoretical modeling of this process. Firstly, since no covalent changes in bonding occur during adsorption, the isotope effects on this process are expected to be very small [33] as in the cases of phase transfer [34] and binding [35]. So the first question is if it is possible to reliably predict their values theoretically, and if so what are the best theory levels to achieve this goal. Secondly, since the orientation of an aromatic compound over the graphene sheet can adopt a number of different orientations, could isotopic fractionation on adsorption be an indicative tool of the actual conformational structure of the graphene–aromatic compound-complex. In this contribution, we address the above questions using benzene as the model compound and a C_54_ graphene sheet.

## 2. Results and Discussion

**Figure 1:**
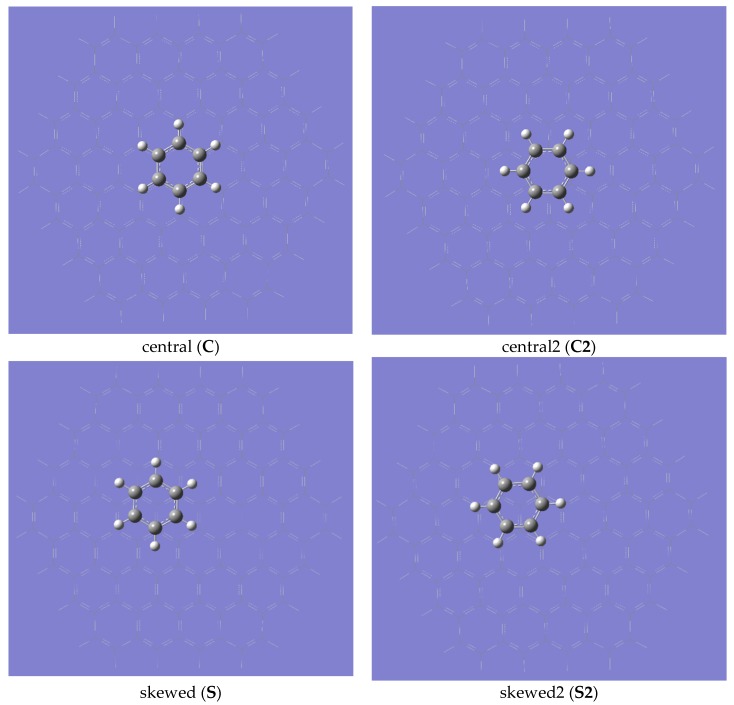
The positions of the benzene ring over the graphene sheet.

Benzene molecules adsorbed over a graphene surface can be oriented in several ways. We have considered the four canonical positions illustrated in Figure 1. In orientation **C** the ring overlays symmetrically central ring of the graphene model with all atoms (both carbons and hydrogens) occupying positions directly over the carbon atoms. Rotation of the benzene ring leads to a symmetrical structure **C2** in which hydrogen atoms are placed over the centers of the ring while the midpoints of the C-C bond are placed over the carbon atoms of the central ring of the graphene model. The translation of the benzene ring along the plane parallel to that of graphene so that one of the carbon atoms of graphene is located directly under the center of the benzene ring yielded the third skewed model (**S**). The subsequent rotation of the benzene ring into a position in which all the hydrogen atoms of benzene overlaid the carbon atoms resulted in model **S2**.

**Figure 2:**
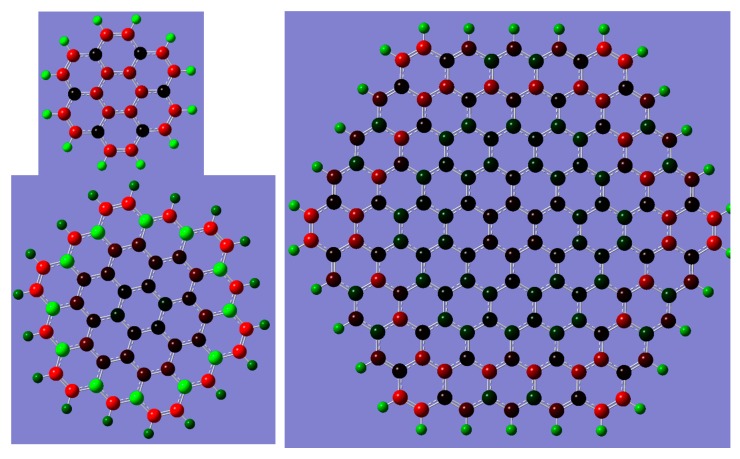
The comparison of Mülliken partial atomic charges. Graphene models C_24_ (upper left), C_54_ (bottom left), and C_150_ (right). Red to black to green indicating values from negative (–0.1e) to neutral to positive (0.1e).

While a few different sizes of the graphene sheet were explored, the main model used in the present studies contains 54 carbon atoms (and is referred to as C_54_) which is a compromise between the computational cost of Hessian calculations and the necessity to consider a sheet large enough to efficiently neglect edge effects. In fact, in the modeling of the adsorption of aromatic compounds on graphene sheets, sizes from C_24_ [28] to C_150_ [29] have been used. In the former case, the edge effects cause the graphene and benzene (and its nitro derivatives) planes to not be parallel. In the latter case, Wang and coworkers have shown that from among the three density functionals suitable for computing the energy of adsorption of benzene (and its fluoroderivatives) on graphene, the ωB97xd [36] functional expressed in the def2-TZVPP basis set [37,38] is least dependent on the size of the graphene model. We have, therefore, decided to use this theory level together with the C_54_ model of the graphene sheet. As illustrated in Figure 2, this model is minimal and the charge distribution should not cause edge effects for the models considered (see Figure 1).

In agreement with the literature data [29], we have found that the **S** structure is the most stable and therefore we have carried the benchmarking of isotopic fractionation calculations for this structure. In fact, in our studies, other initial structures also converged to this one except for the QM/QM calculations. It should be noted, however, that cases where optimization to structures other than **S** was successful, confirming that the energy landscape is very flat with electronic energies of different structures being very close and entropic effects dominating. Thus the most stable structure on the Gibbs free energy surface might be different than that on the potential energy surface (PES). For example, at the M06-2X/def2-SVPP level of theory, **S** is more stable than **S2** by 0.14 kcal/mol, but on the Gibbs free energy surface, the stability is reversed with **S** being more stable by 0.21 kcal/mol.

**Figure 3:**
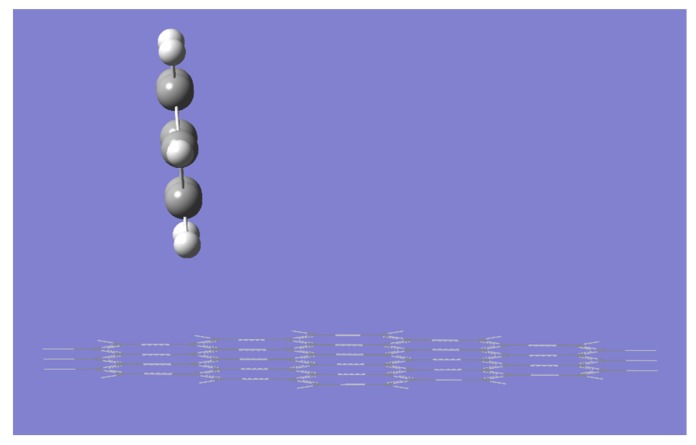
The structure **V** obtained at the DFT/DFTB level of theory.

In all studied theory levels, the distance between the planes, the only significant geometric parameter, was about 3.3 Å in agreement with earlier reports. However, in the case of ONIOM calculations with DFTB as the lower theory level, we were able to optimize only the **S2** structure. In all other attempts, the optimization resulted in the unrealistic structure **V**, presented in Figure 3, in which the angle between planes approaches 90 degrees. Interestingly, in this case, the electronic energy of the **V** structure is lower than that of **S2** by 0.34 kcal/mol, while at the Gibbs free energy surface, **S2** is more stable by 0.23 kcal/mol. Furthermore, we were unsuccessful in optimizing structure **C** to a local minimum at any considered theory level—it seems to be a transition state for the rotation of the benzene ring over the graphene plane.

An isotope effect on adsorption is formally an equilibrium isotope effect. We can describe the adsorption process by the following scheme:

(1) $$B+G\begin{array}{c} K\\ ⇄ \end{array}\mathrm{BG}$$

in which B stands for benzene, G for graphene, and K for the equilibrium constant then, based on the statistical thermodynamics, the corresponding isotope effect (IE) can be described in terms of partition functions [39]. Within the Born–Oppenheimer and Teller–Redlich approximations, it can be expressed solely in terms of pure harmonic vibrational frequencies:

(2) $$\mathrm{IE}=\overset{3n^{B}-6}{\underset{i}{\prod }}\frac{u_{\mathrm{iH}}^{B}\times sinh\left(u_{\mathrm{iL}}^{B}/2\right)}{u_{\mathrm{iL}}^{B}\times sinh\left(u_{\mathrm{iH}}^{B}/2\right)}/\overset{3n^{\mathrm{BG}}-6}{\underset{i}{\prod }}\frac{u_{\mathrm{iH}}^{\mathrm{BG}}\times sinh\left(u_{\mathrm{iL}}^{\mathrm{BG}}/2\right)}{u_{\mathrm{iL}}^{\mathrm{BG}}\times sinh\left(u_{\mathrm{iH}}^{\mathrm{BG}}/2\right)}$$

where *n* is the number of vibrational degrees of freedom (of B and BG), and *u_i_* = *hν_i_/(k_B_T)*, where *ν_i_* are isotopic frequencies, T is absolute temperature, and *h* and *k_B_* are the Planck’s and Boltzmann’s constants, respectively. As already mentioned, the isotope effects on adsorption are small. Therefore, for clarity of the discussion we use isotopic fractionation, ε, which is related to the isotope effect by Equation (3) with units of parts per thousand (called “per-mil”, ‰), i.e., the isotope effect is expressed as an inverse deviation from unity (no isotope effect) multiplied by a thousand [40]:

ε = (1/IE − 1) × 1000(3)

Calculations of isotope effects were performed for fully isotopically substituted reactants (perdeuterated or ^13^C_6_ benzene). Isotopic fractionations per position (ε_perD_ and ε_perC_) were subsequently obtained using the rule of geometric means [41] which describes the additivity of isotope effects (see [33] p. 391 for details).

In the initial calculations, we confirmed that the expected values of IE are small, in the range of parts per thousand or less. Therefore, we studied the influence of the convergence criteria on the final value of the calculated isotope effects. Out of several cases, the most illustrative is a simple test of calculating “isotope effects” where the “substrate” is a molecule converged at a higher threshold, and the “product” is the same molecule converged with tighter convergence criteria. We have calculated two “isotope effects” using the benzene molecule optimized with the convergence criteria (RMS force 0.000300 a.u.—default in Gaussian packages), which is then increased thirty times (0.000010 a.u.), and three hundred times (0.000001 a.u.), using the tight and very-tight options, respectively. Furthermore, we have compared the results obtained using Gaussian versions 09 and 16. The results obtained for perdeuterated benzene (“isotope effects” of C_6_H_6_ vs C_6_D_6_) at the ωB97xD/def2-TZVPP level of theory are listed in Table 1.

The results collected in Table 1 lead to two main conclusions. Firstly, the default convergence threshold cannot be used for calculations of small isotope effects as the error resulting from insufficient convergence is on the level of the studied isotope effect. Even more severe problems may arise from mixing results from different versions of the Gaussian program. This is because the default criteria for grid size and CPHF convergence are different between these versions. In light of the above results, we have carried out our studies using Gaussian 16 exclusively [42]. The BG complex was optimized using a tight convergence threshold while the vtight option was used for the benzene molecule throughout the presented studies.

Table 2 and Table 3 summarize the results obtained for DTF and ONIOM [43] calculations. At the reference ωB97xd/def2-TZVPP level, the isotope effect for the adsorption of fully deuterated benzene is large and normal (larger than unity), while the corresponding carbon isotope effect is inverse (less than unity). As can be seen from Table 2, no such combination of isotope effects was obtained using the 6-31 + G(d,p) basis set regardless of the functional used. Only when triple-zeta 6-311 + G(d,p) was basis set used was the right direction of isotope effects was obtained albeit the values were substantially smaller. Among results obtained using a double-zeta def2 basis set only results obtained with ωB97xd functional are in good agreement with those obtained at the reference level suggesting that ωB97xd/def2-SVPP might be an economic level for calculating isotope effects on adsorption. In order to test the influence of the graphene flake, we carried out calculations using larger model C_96_. The results are highlighted in Table 2 in the third row by using the italic font. As can be seen, there is little influence of increasing the graphene model size on the resulting isotopic fractionation; the results for deuterium are closer to those obtained with the TZ basis sent, while the results for carbon are negligibly lower than those obtained with aid of the C_54_ model. Two other observations are worth noticing; the tendency of the M06-2X functional to substantially overestimate the absolute value of the isotope effect and the reasonable performance of the CAM-B3LYP and LC-BLYP functionals, although they yield the wrong direction of carbon isotopic fractionation.

In the quest for finding the economic theory level for reliable calculations of the isotope effects on adsorption, we have also studied the performance of the QM/QM calculations within the ONIOM scheme using the reference ωB97xd/def2-TZVPP theory level in the description of benzene and the three semiempirical parametrizations in the description of the graphene. As can be seen from the results collected in Table 3, only PM6 parametrization yielded the correct direction of the isotope effects (normal for deuterium and inverse for carbon). The absolute values are, however, significantly underestimated. The calculations with DFTB mostly led to the wrong **V** structure. In both cases of PM6 and PM7, the calculations three canonical forms (except for **C**) were identified as local minima. The results obtained with the latter parametrization suggest that isotopic fractionation can be indicative of the orientation of the benzene molecule over the graphene. This conclusion, however, does not agree with the results obtained using PM6 and since this parametrization yielded results in better agreement with the reference level, we conclude that isotopic fractionation is a rather poor indicator of the geometrical features of the benzene–graphene complex.

The costliest step in the theoretical predictions of isotope effects is the calculation of Hessians. Adsorption seems to be an ideal process to test the performance of the partial Hessian analysis, as the interactions between the absorber and the adsorbed molecule are small (no valence bonds), even more so in the studied system since the charge transfer is minimal and the dominant forces are weak van der Waals interactions. We have, therefore, calculated the isotopic fractionations using the structure optimized at the reference level using Hessian only for a part of the system (benzene molecule in our case) in the field of the point charges of the remaining part (graphene atoms). This protocol is a routine in some programs (e.g., fDynamo [44]) while, in Gaussian, it requires the proper manual preparation of the input. Both Mülliken and ATP charges have been tested. As can be seen from the data collected in Table 4, the results obtained with Mülliken charges are closer to those obtained by a full Hessian analysis. In this same manner, we have also calculated isotopic fractionation for the C_150_ model. The results are slightly smaller than those described above. This might indicate that the employed C_54_ model is still too small and the edge effects contribute artificially to the calculated values. It is, however, hard to test as the calculations with a full Hessian analysis for the C_150_ model are prohibitively time-consuming.

According to the findings of Williams and coworkers [45], a partial Hessian analysis should be performed for 3n rather than 3n-6 degrees of freedom. As can be seen from the results presented in the last two rows of Table 4 in the case of the results obtained using 3n^B^ or 3n^B^-6 degrees of freedom, the adsorption difference between them is negligible.

## 3. Computation Methods

The initial structures of the complexes were prepared using the HyperChem program [46]. Subsequent geometry optimizations and Hessian calculations were performed with the Gaussian package [42], visualized and analyzed using GaussView [47]. No periodic conditions have been used. Convergence criteria are described in the Results and Discussion Section. The following functional basis sets are detailed in Table 2: ωB97xd^34^, B97d3 [48], B97d [49], M06-2X [50], LC-BLYP [51], cam-B3LYP [52], and B3LYP [53,54,55,56] have been tested with def2-TZVPP [35,36], def2-SVPP [35,36] 6-31 + G(d,p) [57,58,59], and 6-311+G(d,p) [55,56,60,61]. The PM6 [62], PM7 [63], and DFTB [64,65] semiempirical parametrizations were used in ONIOM as the lower theory level (real layer). Calculations of vibrations, thermochemistry, and isotope effects assumed the standard gas phase. Our own program for isotope effects calculations, Isoeff17 [66], has been used. This program can be downloaded on an “as is” basis from http://paneth.p.lodz.pl/en/strona-glowna/innowacje/isoeff/.

## 4. Conclusions

**Figure 4:**
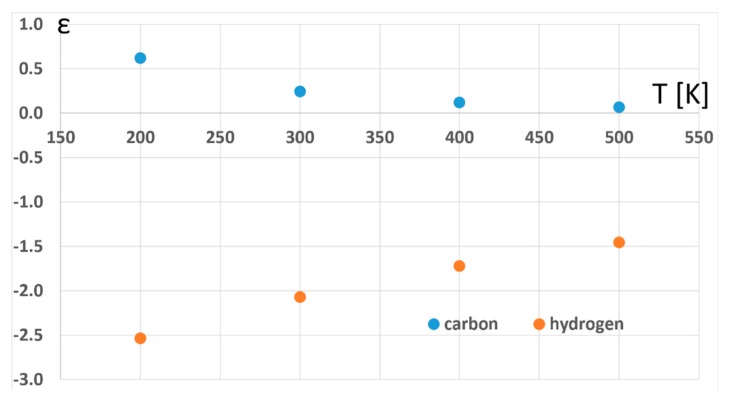
The temperature dependence of the calculated isotopic fractionations.

The main questions addressed in this contribution concern the possibility of using the adsorption of aromatic compounds on graphene for aromatic enrichment and the possibility of using the values of isotope effects for distinguishing between different orientations of the adsorbed molecules. For the studied model compound (benzene), both the differentiation of the adsorbed molecule orientations over the graphene surface using isotopic fractionation, as well as the application of the adsorption on graphene for isotopic enrichment, seem realistic only for the deuterated species due to the small values and small differences of the carbon isotope effects associated with this process. Furthermore, the effects of the temperature are quite small, thus, extremely low temperatures would be needed to increase the isotopic fractionation, as illustrated in Figure 4. It should be noted, however, that the nonpolar nature of the models used in the present studies prevents significant charge transfer which could have made the isotope effects much larger, such as in the case of hydrogen-bonded systems [67]. It is thus possible that the corresponding values of the isotope effects for polar compounds, [68] like nitroaromatics, are substantially larger, rending the process suitable for the practical use of isotopic enrichment. This possibility will be explored in future studies.

In the process, we have explored the applicability of the theoretical predictions of the isotope effects on the adsorption of aromatic compounds on graphene. Our studies indicate that in order to reliably calculate the values of these isotope effects, geometries need to be optimized with convergence limits significantly tighter than those set as default in most of the routinely used programs (Gaussian package in our case). Even with these constraints, different density functionals yield scatter results. Taking ωB97xd/def2-TZVPP as the reference level, we conclude that from among the economic theory levels, only this functional expressed in the def2-SVPP basis set gives acceptable results. In fact, a def2 family of basis sets seems better suited for calculations of isotope effects on adsorption than Pople’s type basis sets although the triple-zeta basis set gives considerably better results. The QM:QM approach within the ONIOM scheme did not yield satisfactory results with the semiempirical parametrizations tested (PM6, PM7, DFTB). The use of partial Hessian, on the other hand, seems to be an attractive alternative since this approach yields acceptable values at an enormous increase of speed.

## Figures and Tables

**Table 1:** The influence of the convergence threshold and version of the program on the error of isotope effect calculations.

Gaussian Version	Convergence Criteria	“IE”	“ε”
G09 rev. E01	default ⇄ tight	1.000374	−0.37
	tight ⇄ vtight	1.000007	−0.01
G16 rev. B01	default ⇄ tight	1.000559	−0.56
	tight ⇄ vtight	1.000007	−0.01
G09 ⇄ G16	default	1.001907	−1.90
	tight	1.002092	−2.09
	vtight	1.002056	−2.05

**Table 2:** The isotope effects (IE) and isotopic fractionations (ε) obtained for S at different levels of theory.

Functional	Basis Set	D_6_-IE	ε_perD_	^13^C_6_-IE	ε_perC_
ωB97xd	def2-TZVPP	1.01674	−2.76	0.99801	0.33
ωB97xd	6-311 + G(d,p)	1.00138	−0.23	0.99901	0.16
ωB97xd ^a^	def2-SVPP	1.01762	−2.91	0.99864	0.23
ωB97xd	def2-SVPP	1.01252	−2.07	0.99853	0.25
B97d3	def2-SVPP	0.97265	4.63	0.99725	0.46
B97d	def2-SVPP	0.97778	3.75	0.99675	0.54
am-B3LYP	def2-SVPP	1.00366	−0.61	1.00094	−0.16
LC-BLYP	def2-SVPP	1.02588	−4.25	1.00091	−0.15
M06-2X	def2-SVPP	1.06471	−10.40	1.00163	−0.27
ωB97xd	6-31 + G(d,p)	0.99595	0.68	0.99944	0.09
B97d3	6-31 + G(d,p)	0.98191	3.05	0.99750	0.42
B97d	6-31 + G(d,p)	0.98998	1.68	0.99770	0.38
cam-B3LYP	6-31 + G(d,p)	1.00441	−0.73	1.00115	−0.19
LC-BLYP	6-31 + G(d,p)	1.02935	−4.81	1.00109	−0.18
M06-2X	6-31 + G(d,p)	1.07768	−12.39	1.00151	−0.25
B3LYP	6-31 + G(d,p)	0.99871	0.21	1.00098	−0.16

^a^ results obtained for the C_96_ model.

**Table 3:** The isotope effects (IE) and isotopic fractionations (ε) obtained using ONIOM QM:QM two-layer model and ωB97xd/def2-TZVPP for benzene.

:QM	Structure	D_6_-IE	ε_perD_	^13^C_6_-IE	ε_perC_
-	**S**	1.01674	−2.76	0.99801	0.33
PM7	**C2**	0.97754	3.79	0.99918	0.14
PM7	**S**	0.99944	0.09	0.98089	3.22
PM7	**S2**	0.97889	3.56	0.99964	0.06
DFTB	**V**	1.00969	−1.61	1.00032	−0.05
DFTB	**S2**	1.00011	−0.02	1.00038	−0.06
PM6	**C2**	1.00146	−0.24	0.99954	0.08
PM6	**S**	1.00112	−0.19	0.99987	0.02
PM6	**S2**	1.00412	−0.69	0.99997	0.01

**Table 4:** The isotope effects (IE) and isotopic fractionations (ε) obtained using partial Hessian analysis at the ωB97xd/def2-TZVPP level of theory.

Graphene Model	Degrees of Freedom	Point Charges	D_6_-IE	ε_perD_	^13^C_6_-IE	ε_perC_
C_54_	3n^BG^-6	-	1.01674	−2.76	0.99801	0.33
C_54_	3n^B^-6	Mülliken	1.01533	−2.53	0.99993	0.01
C_54_	3n^B^-6	APT	1.00681	−1.13	1.00025	−0.04
C_150_	3n^B^-6	Mülliken	1.01088	−1.80	1.00019	−0.03
C_150_	3n^B^	Mülliken	1.01074	−1.78	1.00013	−0.02
